# Sarcoid-like reactions: a potential pitfall in oncologic imaging

**DOI:** 10.1007/s00259-020-04960-2

**Published:** 2020-07-24

**Authors:** Selamawit Gebrekidan, Tina Schaller, Andreas Rank, Malte Kircher, Constantin Lapa

**Affiliations:** 1 Nuclear Medicine, Medical Faculty, University of Augsburg, Augsburg, Germany; 2 General Pathology and Molecular Diagnostics, Medical Faculty, University of Augsburg, Augsburg, Germany; 3 Internal Medicine II, Medical Faculty, University of Augsburg, Augsburg, Germany

A 44-year-old female with the primary diagnosis of classic (nodular sclerosing) Hodgkin’s lymphoma (HL) was referred for further diagnostic work-up. Whole-body positron emission tomography/computed tomography (PET/CT) using ^18^F-fluorodeoxyglucose (FDG) revealed multiple nodal HL manifestations in the right external iliac and inguinal regions (Ann Arbor II, red arrow).

Subsequently, the patient underwent chemotherapy with two cycles of escalated BEACOPP, resulting in CT-based partial response. Therefore, two cycles of ABVD and local radiotherapy were added in curative intent.

While the formerly affected lymph nodes showed a complete metabolic response at end-of-treatment PET/CT (Deauville score: 2), newly enlarged mediastinal and bilateral hilar lymph nodes with increased FDG-uptake as well as several hypermetabolic bone lesions were detected (red stars, insert B–D). A biopsy from a tracer-avid lesion in the anterior superior iliac spine was obtained. Histology revealed only non-caseating epitheloid cells and giant cells (black stars, insert A) adjacent to blood vessels (black arrows, insert A), indicative of sarcoidosis. Since bronchoalveolar lavage and endobronchial ultrasound-guided transbronchial needle aspiration did not reveal malignant cells either, no further therapy was initiated. Due to the absence of clinical symptoms, a sarcoid-like reaction was favored over actual sarcoidosis as the possible diagnosis.

After 3 months, follow-up PET/CT showed almost complete remission of the lymphadenopathy and bone lesions (Deauville score: 2) supporting the hypothesis of a tumor-related sarcoid-like reaction.

Sarcoid-like reactions are observed in up to 13.8% of HL patients and should be taken into account as a differential diagnosis for hilar and/or mediastinal lymphadenopathy in patients with a history of malignancy [1–3]. In case of uncertainty, a tissue biopsy is recommended to differentiate between the two entities and to avoid misdiagnosis.


